# Serum levels of specific IgE to *Staphylococcus aureus* enterotoxins in patients with chronic rhinosinusitis

**DOI:** 10.3892/etm.2015.2247

**Published:** 2015-02-02

**Authors:** XIN-YAN CUI, JIAN-LIANG MIAO, HAN-QIANG LU, QIN-HONG QI, XI CHEN, JIN XU, ZI-PING LIN, ZHI-BIN CHEN, MIN YIN, LEI CHENG

**Affiliations:** 1 Department of Otorhinolaryngology, The First Affiliated Hospital, Nanjing Medical University, Nanjing, Jiangsu 210029, P.R. China; 2 Department of Otorhinolaryngology, The Fifth Affiliated Hospital, Yangzhou University, Changshu, Jiangsu 215500, P.R. China; 3 Department of Otorhinolaryngology, Affiliated Hospital, Jiangsu University, Zhenjiang, Jiangsu 212001, P.R. China; 4 Allergy Laboratory, The First Affiliated Hospital, Nanjing Medical University, Nanjing, Jiangsu 210029, P.R. China; 5 International Center for Allergy Research, Nanjing Medical University, Nanjing, Jiangsu 210029, P.R. China

**Keywords:** sinusitis, nasal polyps, *Staphylococcus aureus*, superantigens, immunoglobulin E, eosinophil cationic protein

## Abstract

The aim of this study was to determine the prevalence of *Staphylococcus aureus* enterotoxins (SEs) in the serum from patients with chronic rhinosinusitis (CRS) and its involvement in the condition. Thirty CRS patients without nasal polyps (CRSsNP), 40 CRS patients with nasal polyps (CRSwNP), and 30 healthy controls were enrolled in this study. Peripheral blood was obtained and analyzed to measure the serum levels of total IgE, specific IgE to SEA, SEB and SEC, and eosinophil cationic protein (ECP) using ImmunoCAP assays. The positive rate and level of serum specific IgE to SEB, but not to SEA or SEC, were significantly higher in CRSwNP patients compared with the controls (P=0.027 and P=0.021, respectively). No significant differences were found between CRSsNP patients and controls, or between CRSsNP and CRSwNP patients. Serum total IgE was significantly elevated and positively correlated with SEB-specific IgE in the CRSsNP (P<0.001; r=0.393, P=0.032) and CRSwNP (P<0.001; r=0.581, P<0.001) groups. ECP was also significantly increased in the CRSsNP (P=0.002) and CRSwNP (P<0.001) groups, but not correlated with specific IgE to SEs in either CRS group. The results suggest that SEB may play a role in the pathogenesis of CRSwNP.

## Introduction

Chronic rhinosinusitis (CRS) is characterized by mucosal inflammation of the nose and paranasal sinuses and often taken as an umbrella term for a heterogeneous group of sinus diseases. CRS has been divided into CRS without nasal polyps (CRSsNP) and with nasal polyps (CRSwNP) on the basis of clinical presentation (1,2). However, controversy exists as to whether they represent the different stages of one disorder or separate entities due to their distinct histomorphology, inflammatory and remodeling profiles (3).

Over the last decades, mounting evidence has suggested that the etiology and pathophysiology of CRS is complex and multifactorial (4). Of the multiple etiological hypotheses, including bacterial, fungal, microbial biofilm, superantigen and immune barrier hypotheses (4–6), an exogenous pathogen is probably essential for the development and persistence of mucosal inflammation. *Staphylococcus aureus* enterotoxins (SEs), secreted by *Staphylococcus aureus* (*S. aureus*), the most common colonizer of nasal passages and sinuses, are broadly classified as superantigens (7). It has been speculated that SEs may aggravate inflammation severity in airway diseases, such as CRS, asthma and allergic rhinitis (8–10).

Although a causal relationship of *S. aureus* in patients with CRS has not been established, SEs might skew the cytokine response towards a Th2 phenotype inducing both eosinophilia and the production of polyclonal IgE (11), and thus contribute to, at least in some cases, the development of CRSwNP (8,12,13). By contrast, the evidence for the effect of SEs in CRSsNP patients is so far relatively lacking (14). Recent studies have shown that the inflammatory phenotypes in Chinese patients with CRS are inconsistent with those in Caucasian subjects despite their similar histomorphological pattern (15–17). Further understanding the role of SEs in Chinese subjects with CRS may help to provide insight into the mechanistic basis underlying CRS. In the present study, the serum levels of total IgE, specific IgE to SEA, SEB, and SEC, and eosinophil cationic protein (ECP) were investigated in Chinese patients with CRSsNP and CRSwNP.

## Materials and methods

### Study subjects

A total of 70 patients undergoing endoscopic sinus surgery for CRS were enrolled consecutively at the Department of Otorhinolaryngology, the First Affiliated Hospital, Nanjing Medical University (Nanjing, China). The diagnosis of CRS was based on medical history, clinical symptoms, endoscopic examination, and sinus CT scanning according to the European Position Paper on Rhinosinusitis and Nasal Polyps 2007 as well as the Chinese CRS guidelines (1,2). Thirty of the patients were classified as CRSsNP, and the other 40 patients were CRSwNP. The study also involved 30 healthy volunteers with no sinonasal diseases as control subjects. Atopic status was evaluated by screening for specific IgE to common aeroallergens (Phadiatop; Phadia AB, Uppsala, Sweden). Subjects who had taken glucocorticoids within 4 weeks, H1-antihistamines or leukotriene modifiers within 2 weeks, and/or had asthma, atopic dermatitis or Samter’s triad were excluded. The characteristics of the CRS patients and healthy controls are shown in Table I. This study was approved by the ethics committee of Nanjing Medical University, and all participants gave their written informed consent.

### Measurement of total IgE, specific IgE and ECP in serum

Peripheral blood (3 ml) was collected from each subject. After centrifugation at 100 × g for 10 min, the serum was separated and stored at −70°C until further analysis. The levels of total IgE, specific IgE to SEA, SEB and SEC, and ECP in sera were measured using ImmunoCAP assays (Phadia AB) according to the manufacture’s recommendations. The detection limits were set at <2 kU/l for total IgE and 2 μg/l for ECP. A concentration of specific IgE ≥0.35 kUA/l was considered as positive, and the levels were expressed as the following grades: 0, <0.35 kUA/l; I, 0.35–0.69 kUA/l; II, 0.7–3.49 kUA/l; III, 3.5–17.49 kUA/l. IV, 17.5–49.9 kUA/l; V, 50–100 kUA/l; VI, >100 kUA/l.

### Statistical analysis

Statistical analyses were performed using SAS software version 9.1.3 (SAS Institute, Cary, NC, USA). Specific IgE to SEA, SEB, and SEC were analyzed as ordinal data. Total IgE and ECP are presented as median and interquartile range. Data were first compared within different groups by the Kruskal-Wallis H test. The Mann-Whitney U test with the Bonferroni’s post hoc test was then applied to evaluate the statistical differences between-group comparison. Differences in proportions among groups were compared with the χ^2^ test or Fishers’ exact test. Correlations were calculated using the Spearman test. A P-value <0.05 was considered statistically significant.

## Results

### Serum SE-specific IgE in the CRS and control groups

**Figure 1:**
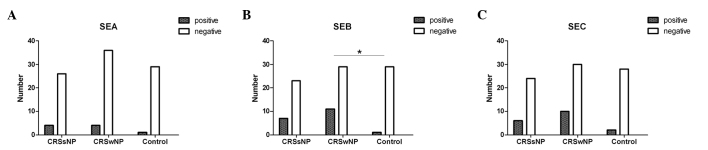
Number of samples that were positive (dark bars) and negative (light bars) for serum specific IgE to (A) SEA, (B) SEB and (C) SEC measured by ImmunoCAP assays among 30 CRSsNP patients, 40 CRSwNP patients and 30 controls. ^*^P<0.05. SEA, *Staphylococcus aureus* enterotoxin A; SEB, *Staphylococcus aureus* enterotoxin B; SEC, *Staphylococcus aureus* enterotoxin C; CRSsNP, chronic rhinosinusitis without nasal polyps; CRSwNP, chronic rhinosinusitis with nasal polyps.

As shown in Table I, specific IgE against at least one subtype of SEs was detected in the serum from 9 of 30 (30.0%) subjects with CRSsNP, 13 of 40 (32.5%) with CRSwNP and 2 of 30 (6.7%) controls, respectively (level range, grades I-III). Of these, only the positive rate of SEB-specific IgE was significantly higher in the CRSwNP group than that in the control group (P=0.027; Fig. 1). Furthermore, the serum level of specific IgE to SEB, rather than that to SEA and SEC, was elevated markedly in the CRSwNP group compared with that in the control group (P=0.021). The positive rate and level of SEB-specific IgE in CRSsNP group showed an increasing trend but did not reach significance (P=0.06 and P=0.069, respectively).

### Serum total IgE and ECP in the CRS and control groups

**Figure 2:**
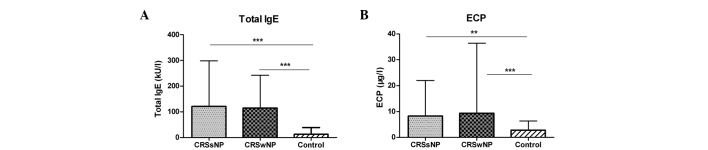
Serum levels of (A) total IgE and (B) ECP measured by ImmunoCAP assays among 30 CRSsNP patients, 40 CRSwNP patients and 30 controls. ^**^P<0.01; ^***^P<0.001. ECP, eosinophil cationic protein; CRSsNP, chronic rhinosinusitis without nasal polyps; CRSwNP, chronic rhinosinusitis with nasal polyps.

As shown in Fig. 2, the serum levels of total IgE were significantly higher in the CRSsNP and CRSwNP groups than those in the control group (both P<0.001). Also, the serum levels of ECP were elevated markedly in the CRSsNP group (P=0.002) and in the CRSwNP group (P<0.001) compared with those in the control group. However, no significant differences were observed between the two CRS groups.

### Correlation between SE-specific IgE and total IgE/ECP in the CRS groups

In the CRSsNP group, serum total IgE was positively correlated with specific IgE to SEA (r=0.470, P=0.009), to SEB (r=0.393, P=0.032) and to SEC (r=0.397, P=0.03). In the CRSwNP group, serum total IgE was also positively correlated with specific IgE to SEB (r=0.581, P<0.001) and to SEC (r=0.501, P=0.001), but not to SEA. There was no correlation between the serum levels of ECP and specific IgE to SEs in either group of CRS. The results are summarized in Table II.

## Discussion

The SEs have been described as superantigens due to their ability to bridge major histocompatibility (MHC) class II molecules and directly activate polyclonal T cells and B cells in a nonspecific manner (18,19). To date, there are >20 distinct SEs including SEA through V and toxic shock syndrome toxin-1 (TSST-1), but only a few of them have been well researched (7).

In the present study, the serum specific IgE antibodies against three common staphylococcal superantigens (SEA, SEB and SEC) were detected in patients with CRSsNP and CRSwNP, and healthy controls. The results demonstrated that the positive rate and level of serum specific IgE to SEB in CRSwNP patients were significantly higher in comparison with those in the controls. In line with earlier findings (20–22), the results of the present study have revealed that specific IgE to SEs can be detected in serum, and may have an effect on CRSwNP. SEs are known to have the ability to cross airway epithelial barriers in an immunologically intact form (23,24), possibly via inducing extensive inflammation that brings about an increase in epithelial permeability and a reduction in tight junction proteins (7). In addition, as reported in an *in vivo* study in mice by Hamad *et al* (25), SEB is more efficient at traversing the epithelial barrier and entering the blood than SEA, although both rapidly reached functional levels in the serum following oral administration. This may account for the discrepancy of serum levels among different specific IgE against SEs.

Previous studies have suggested that SEB stimulation highly increases the expression of TNF-α, IFN-γ, IL-2, IL-4, IL-5, IL-13, and IL-17 (11,26). These cytokines are capable of promoting the recruitment of neutrophils or eosinophils by means of induction of chemokines and granulopoiesis factors. SEB is likely to be able to induce multiple T-effector cell cytokines, leading to a mixed inflammation pattern involving the infiltration of neutrophilic and eosinophilic granulocytes. Furthermore, a mixed Th1/Th2/Th17 pattern has been confirmed in Chinese CRSsNP and CRSwNP patients (15). Accordingly, SEB may play a comparable role in the two CRS entities of Chinese patients. However, a direct association was not observed between SEB and CRSsNP in the present study, although specific IgE against SEB tended to be higher in CRSsNP patients than in the controls.

Significant increases of total IgE and ECP levels in the sera from the two CRS groups were observed in the present study, and an analysis of the correlation among total IgE, ECP and specific IgE to SEs was conducted. Total IgE had a positive correlation with specific IgE to SEB as well as to SEC in CRSwNP, and these correlations were stronger than those in CRSsNP. Kowalski *et al* (9) found that total IgE had a strong correlation with specific IgE to SEs in serum from asthma patients that was independent of atopic status, and these two factors significantly correlated with asthma severity markers. Accordingly, total IgE may be directly promoted by specific IgE to SEs; in addition, they both have predictive roles in the development and persistence of airway inflammation. By contrast, inconsistent with findings in Caucasian patients (20), no significant correlation was detected between ECP and specific IgE to SEs in the CRSwNP or CRSsNP groups in the present study. Patients with asthma and atopic dermatitis were excluded, and the prevalence of allergic rhinitis was not different among groups. The increased levels of ECP, which indicate an intense activation of eosinophilic inflammation in the sera from the two CRS groups seem to be unassociated with atopic status in this study. However, various effects of unknown factors on peripheral blood should be taken into account, and the relationships require further study in local tissue. In view of the considerable differences in inflammatory pattern between Chinese and Caucasian patients, SEs might have different effect on sinonasal inflammation via varying immune system responses.

It may be speculated that *S. aureus* and SEB formed in the sinonasal tissues might be responsible for participating and amplifying the development of mucosal inflammation, and further be a source of persistent inflammation due to the capacity of *S. aureus* for residing in the nonphagocytic eukaryotic cells and escaping from immune surveillance (27). However, direct evidence for this and the exact molecular mechanisms by which *S. aureus* and SEs exert their effect in CRS require exploration in future studies.

The limitation of the present study is that the levels of specific IgE to SEs in the local tissues, sinonasal mucosa and polyps were not measured due to the lack of sufficient samples from control subjects. In further studies, it is recommended that tissue samples should also be analyzed and comparative and correlation analysis with serum expression conducted to elucidate the role of SEs (particularly SEB) in the development and severity of CRS comprehensively.

In summary, the positive rate and level of SEB-specific IgE were significantly higher in the serum from the Chinese CRSwNP patients than in that from the healthy controls. In addition, the presence of total IgE correlated positively with that of SEB-specific IgE. It is suggested that SEB may play a role in the pathogenesis of CRSwNP in Chinese patients.

## Figures and Tables

**Table I:** Characteristics and laboratory data of the CRS patients and healthy controls.

Variable	CRSsNP (n=30)	CRSwNP (n=40)	Control (n=30)	P-value^a^
Gender, male:female	16:14	28:12	15:15	>0.05
Age, years	45.2±15.6	46.1±12.6	40.6±10.2	>0.05
Atopy	2/30	4/40	1/30	>0.05
Total IgE, kU/l	121.5 (64.6–298.5)^b^	114.5 (54.4–242.3)^b^	12.0 (5.14–38.1)	<0.001
ECP, μg/l	8.18 (4.53–22)^c^	9.31 (5.46–36.4)^b^	2.71 (<2–6.27)	<0.001
SE-specific IgE, positive number (%)				
SEA, grade I/II/III	2/2/0 (13.3)	4/0/0 (10.0)	1/0/0 (3.33)	>0.05
SEB, grade I/II/III	5/1/1 (23.3)	3/8/0 (27.5)^d^	1/0/0 (3.33)	0.025 (0.018)
SEC, grade I/II/III	1/4/1 (20.0)	4/5/1 (25.0)	2/0/0 (6.67)	>0.05
Positive SEs	9/30	13/40^d^	2/30	0.028

Age is shown as mean ± SD; total IgE and ECP are presented as the median (interquartile range).

a Compared among the CRSsNP, CRSwNP and control groups.

b P<0.001,

c P<0.01,

d P<0.05, compared with the controls.

CRS, chronic rhinosinusitis; CRSsNP, CRS without nasal polyps; CRSwNP, CRS with nasal polyps; ECP, eosinophil cationic protein; SE, *Staphylococcus aureus* enterotoxin; SEA, *Staphylococcus aureus* enterotoxin A; SEB, *Staphylococcus aureus enterotoxin* B; SEC, *Staphylococcus aureus* enterotoxin C.

**Table II:** Correlation between SE-specific IgE and total IgE/ECP in sera from CRS patients.

Variable	SEA	SEB	SEC
CRSsNP			
Total IgE	r=0.470, P=0.009	r=0.393, P=0.032	r=0.397, P=0.03
ECP	r=0.069, P=0.718	r=−0.157, P=0.407	r=−0.045, P=0.812
CRSwNP			
Total IgE	r=0.240, P=0.135	r=0.581, P<0.001	r=0.501, P=0.001
ECP	r=−0.088, P=0.590	r=−0.134, P=0.411	r=−0.303, P=0.057

CRS, chronic rhinosinusitis; CRSsNP, CRS without nasal polyps; CRSwNP, CRS with nasal polyps; ECP, eosinophil cationic protein; SE, *Staphylococcus aureus* enterotoxin; SEA, *Staphylococcus aureus* enterotoxin A; SEB, *Staphylococcus aureus* enterotoxin B; SEC, *Staphylococcus aureus* enterotoxin C.
